# Autophagy-Modulating Long Non-coding RNAs (LncRNAs) and Their Molecular Events in Cancer

**DOI:** 10.3389/fgene.2018.00750

**Published:** 2019-01-14

**Authors:** Md Zahirul Islam Khan, Shing Yau Tam, Helen Ka Wai Law

**Affiliations:** Department of Health Technology and Informatics, Faculty of Health and Social Sciences, The Hong Kong Polytechnic University, Hong Kong, Hong Kong

**Keywords:** autophagy, long non-coding RNAs, cancer, therapy, biomarkers

## Abstract

Cancer is a global threat of health. Cancer incidence and death is also increasing continuously because of poor understanding of diseases. Although, traditional treatments (surgery, radiotherapy, and chemotherapy) are effective against primary tumors, death rate is increasing because of metastasis development where traditional treatments have failed. Autophagy is a conserved regulatory process of eliminating proteins and damaged organelles. Numerous research revealed that autophagy has dual sword mechanisms including cancer progressions and suppressions. In most of the cases, it maintains homeostasis of cancer microenvironment by providing nutritional supplement under starvation and hypoxic conditions. Over the past few decades, stunning research evidence disclosed significant roles of long non-coding RNAs (lncRNAs) in the regulation of autophagy. LncRNAs are RNA containing more than 200 nucleotides, which have no protein-coding ability but they are found to be expressed in most of the cancers. It is also proved that, autophagy-modulating lncRNAs have significant impacts on pro-survival or pro-death roles in cancers. In this review, we highlighted the recently identified autophagy-modulating lncRNAs, their signaling transduction in cancer and mechanism in cancer. This review will explore newly emerging knowledge of cancer genetics and it may provide novel targets for cancer therapy.

## Introduction

**FIGURE 1:**
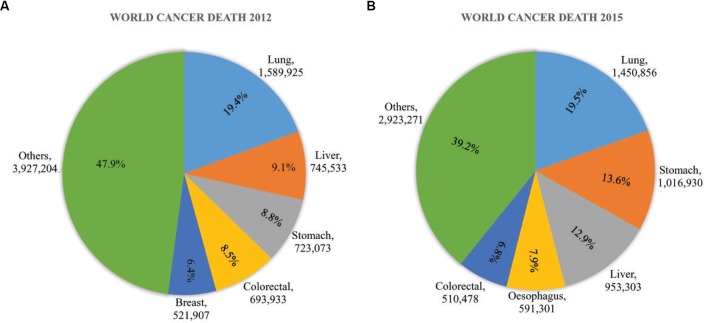
Global cancer impact in **(A)** 2012 and **(B)** 2015. In 2012, the death toll 8.2 million from 14 million new cases. It raised gradually to 8.8 million in 2015. The growth of cancer death was more than 7% from 2012 to 2015. The most common cancers were Lung cancer, liver cancer, stomach cancer, colorectal cancer, esophageal cancer, and breast cancer was a major threat of women cancer death worldwide. These figures were based on data published by WHO and GLOBOCAN (Ferlay et al., 2015; GBD 2015 Risk Factors Collaborators, 2016; WHO, 2018).

Cancer is a global threat of public health because of its high mortality rate. More than 14 million of new cancer cases and ∼60% of death were accounted in 2012 by GLOBOCAN data (Figure 1A). It is also assumed that more than 20 million new cancer cases will be counted in the year 2025 (Ferlay et al., 2015). Cancer death is increasing day by day, 8.8 million death were counted in 2015 where abundant death was reduced in low to moderate income countries (Figure 1B) (GBD 2015 Risk Factors Collaborators, 2016; WHO, 2018). The cancer cases in 10 Asian countries are also estimated to increase from in 6.1 million to 10.7 million in the year 2008 to 2030 respectively. The consecutive death was estimated to raise from 4.1 million to 7.5 million (Sankaranarayanan et al., 2014).

Metastatic tumors are the leading cause of death and pose a great challenge for cancer treatments. Although the molecular basis of carcinogenesis is different in different cancers, the metastasis developmental process is almost similar in all solid tumor cells (Bogenrieder and Herlyn, 2003). The aim of traditional treatments of cancer is either removing or destroying cancerous cells by surgery, radiation therapy, chemotherapy and sometimes personalized treatment. Theoretically, these traditional treatments are very promising but practically none of these are able to cure effectively and efficiently because of poor understating of mechanisms and development of metastasis (Wang Z. et al., 2016).

**FIGURE 2:**
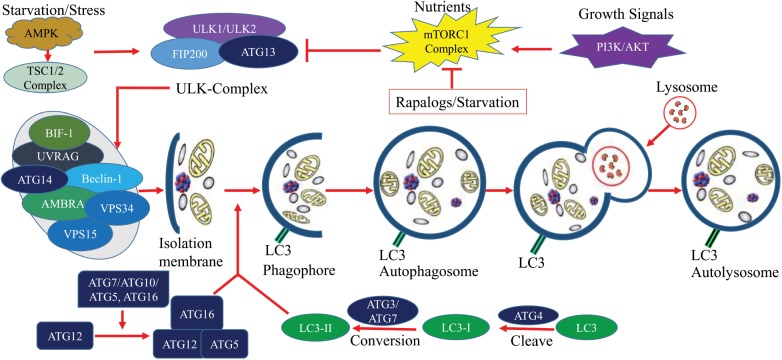
The molecular signaling of the autophagy process. Autophagy is a critical process of cells, it can be simplified into several steps including, Formation of isolation membrane; Nucleation of phagophore; Maturation into autophagosome and Autolysosome. Under starvation/stress, ATP/AMP activates AMPK which further activates TSC1/2 complex. This complex induces autophagy through the blocking of mTOR. Elongation is controlled by ATG7 and ATG10 mediated covalent conjugation with ATG12 and ATG5. ATG5/ATG12 non-covalently bind with ATG16 to form an ATG12/ATG5/ATG16 complex which subsequently adds LC3 to phagophoric barrier. Circular autophagosome formation is carried out by recycling of LC3 (conversion of LC3I to LC3II by ATG4). Mature autophagosome gradually fuses with lysosome for selective degradation and recycling of nutrients. This figure was based on published data (Kim and Lee, 2014; Cicchini et al., 2015; Ktistakis and Tooze, 2016).

Autophagy is a highly conserved and critical regulatory process for cells to maintain homeostasis by lysosomal degradation of various proteins and damaged organelles. Dynamic roles of autophagy have been identified in cancers where it participates in cancer progression, prevention, as well as, drug resistance mechanisms (Santana-Codina et al., 2017). There are three types of autophagy: macroautophagy, microautophagy, and chaperone-mediated autophagy. Macroautophagy is sometimes also referred to as autophagy which is the major autophagic pathways and most extensively studied compared to microautophagy and chaperone-mediated autophagy. In macroautophagy, phagophore is initially formed and matured to autophagosome. Subsequently, autophagosome fused with a lysosome to degrade the internal materials in autolysosome (Figure 2) (Mizushima and Komatsu, 2011). Although autophagy may suppress tumors (Kung et al., 2011) in most cases, the induction of autophagy promotes tumorigenesis by providing survival capability of tumor under microenvironmental stress (Kung et al., 2011; Avalos et al., 2014; White, 2015). Autophagy promotes cancer by inhibiting tumor suppressor protein p53 and controlling the metabolism of cells (Amaravadi et al., 2016). Cellular metabolism and homeostasis are encoded by more than 30 ATGs, their translational products and transduction of signals (Figure 2) (Kim and Lee, 2014; Cicchini et al., 2015; Ktistakis and Tooze, 2016). Tumorigenesis of both benign and malignant tumors are controlled by either single or group of ATG genes (Blessing et al., 2017; Mowers et al., 2017). Thus, in most of the cancers, autophagy is key therapeutic choices in clinical trials (Mowers et al., 2016).

There is increasing evidence that a large number of ncRNAs are actively transcribed from the human genome, controlling diverse cellular metabolic process in growth and development of cancers through regulating various gene expressions (Lin and He, 2017). Most diverse ncRNAs, specifically lncRNAs, are largely involved in cancer initiation, maturation, metastasis and resistance against chemotherapy (Lin and He, 2017; Hu et al., 2018). LncRNAs are >200 nucleotides in length and are mostly transcribed by RNA polymerase II. According to GENCODE (version 18), 13,562 lncRNAs have been identified (Huang X. et al., 2017). LncRNAs have independent expressions in different tissues and different cancers and are responsible for various cancers including BRC, CRC, GC, glioma, HCC, leukemia, LVC, LC, OVC, PTC, and retinoblastoma (He et al., 2016; Huang J. et al., 2017; Huang X. et al., 2017; Li et al., 2017b,d,e; Luo et al., 2017; Ma et al., 2017d; Jiang et al., 2018; Li H. et al., 2018).

Over the past decades, advance research evidence has shown that lncRNAs regulate most of the cancers by means of controlling the autophagy process and modulating the transcriptional and post-transcriptional ATGs (Mowers et al., 2017; Zhang J. et al., 2017). In this review, we aim to provide an overview of recently identified autophagy-modulating lncRNAs autophagy in cancers, their mechanisms and future directions on therapeutic intervention.

**FIGURE 3:**
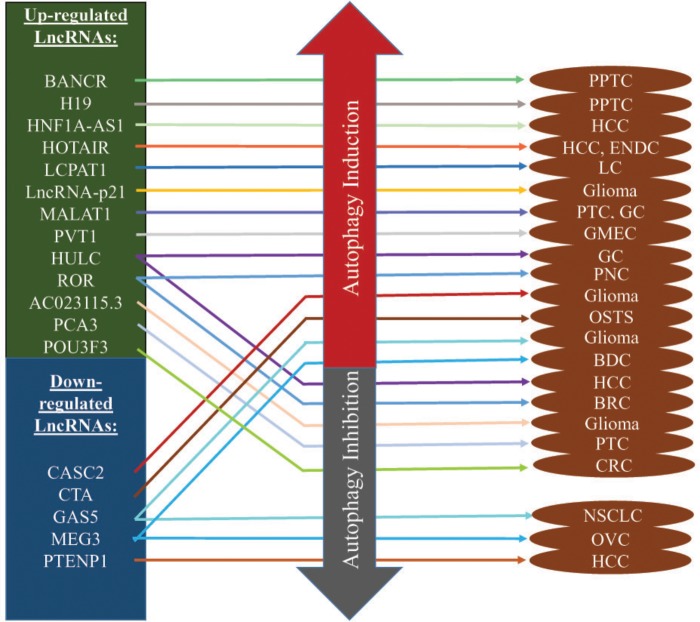
Autophagy-modulating lncRNAs in cancer. Most of the lncRNAs are overexpressed and they induce autophagy to promote/inhibit carcinogenesis. Some of them play key roles in drug resistance while some inhibit autophagy to maintain homeostasis in cancer micro-environments. In the contrary, down-regulated lncRNAs induce/suppress autophagy to promote/inhibit cancer progression.

The field of targeted therapy is rapidly developing with advanced genetic study and their successful applications. Previously considered junk molecules such as lncRNAs are now extensively studied to be established as novel diagnostic biomarkers and therapeutic targets. The biological and physiological roles of autophagy-modulating lncRNAs in carcinogenesis are being unveiled recently. The expression of lncRNAs greatly impacts on the extent of autophagy at different carcinogenic stages, mostly in advanced metastatic stages. A number of research articles suggested that lncRNAs induce or suppress autophagy through ATGs and their signaling pathways (Table 1). The complex process of autophagy modulation by expressions of lncRNAs may suppress or promote carcinogenesis under diverse physiological conditions (Figure 3). Here we described below the recently characterized lncRNAs and their mechanisms through inducing or inhibiting autophagy in different cancers. We also summarized some autophagy-modulating lncRNA which worth further investigation in the field of cancer.

**Table 1:** List of autophagy-modulating lncRNAs and their roles in various cancer.

LncRNAs	Expression in cancer	Autophagy modulation	Functions	Sources	Reference
AC023115.3	Up-regulated	Inhibit autophagy	Increase cisplatin -mediated apoptosis in glioma	Glioma cell lines	Ma et al., 2017b
BANCR	Up-regulated	Induce autophagy	Promote PPTC proliferation, growth and reduce cell population at G1 phase.	Cell line and tumor samples	Wang et al., 2014
CASC2	Down-regulated	Induce autophagy	Glioma progression and temozolomide resistance through controlling mTOR	Glioma cells, tissues and peritumoral brain edema tissues	Jiang et al., 2018
CTA	Down-regulated	Induce autophagy	Promote TNM, larger tumor depth and reduced chemosensitivity in OSTS	OSTS cells and tissues (without pre-operative therapy)	Wang Z. et al., 2017
GAS5	Down-regulated	Induce autophagy/inhibit autophagy	Progression of NSCLC and cisplatin resistance; progression of glioma and cisplatin resistance by inducing autophagy	NSCLC cell lines and stage III tumor tissues; Glioma cell lines	Zhang et al., 2016; Huo and Chen, 2018
H19	Up-regulated	Induce autophagy	Promote PPTC pathogenesis by promoting proliferation	PPTC cells and tissues	Li M. et al., 2018
HNF1A-AS1	Up-regulated	Induce autophagy	Promote TNM, increase growth and apoptosis in HCC	HCC cells and surgically resected tissues	Liu et al., 2016
HOTAIR	Up-regulated	Induce autophagy	Cancer progression and drug resistance in HCC and ENDC	HCC cells and tissues; ENDC cell lines	Yang et al., 2016; Bao et al., 2017; Sun et al., 2017
HULC	Up-regulated	Induce autophagy/ Inhibit autophagy	Promote GC proliferation, migration, and invasion, and reduced apoptosis by inducing autophagy; chemosensitivity of HCC is promoted by inhibiting autophagy	GC cells and tissues; HCC cell lines and tissues	Zhao et al., 2014; Xiong et al., 2017
LCPAT1	Up-regulated	Induce autophagy	Promote carcinogenesis of LC and knockdown gradually reduce *in vivo* tumor size	LC cell lines and patients sample	Yu et al., 2018
LncRNA-p21	Up-regulated	Induce autophagy	Promote proliferation, motility, reduce apoptosis and radio-sensitivity in hepatoma and glioma	Glioma cells and urine samples	Işın et al., 2015; Shen et al., 2017
MALAT1	Up-regulated	Induce autophagy	Promote cell proliferation, metastasis, and autophagy induced drug resistance in MM, PTC, and GC	MM cell lines; PNC cells and pancreatic ductal adenocarcinoma tissues; GC cell lines	Li L. et al., 2016; Yuan et al., 2016; Fu et al., 2017; Gao D. et al., 2017; Huang J. et al., 2017; YiRen et al., 2017
MEG3	Down-regulated	Induce autophagy/inhibit autophagy	Reduced expression promote BDC proliferation, colony formation, and progression through inducing autophagy; while upregulation lead to OVC proliferation and progression is raisedvia inhibiting autophagy	BDC cells and tissues; Epithelial OVC cell and tissues with different stages	Ying et al., 2013; Xiu et al., 2017
PCA3	Up-regulated	Inhibit autophagy	Increase proliferation, migration, and invasion in PTC	PTC cells and tissues	He et al., 2016
POU3F3	Up-regulated	Inhibit autophagy	Promote CRC proliferation and migration	CRC cells and tissues	Shan et al., 2016
PTENP1	Down-regulated	Inhibit autophagy	Promote proliferation and migration in HCC	HCC cell lines	Chen et al., 2015
PVT1	Up-regulated	Induce autophagy	Promote GMEC proliferation migration and angiogenesis	Cerebral microvascular endothelial cell line	Ma et al., 2017c
ROR	Up-regulated	Inhibit autophagy/induce autophagy	Promote proliferation, invasion, migration, tamoxifen resistance and reduced gemcitabine- induced cell death in BRC; up-regulated in PNC to reduce chemosensitivity	BRC cells and tissues (without any therapy); PNC cells and tissues	Chen Y.M. et al., 2016; Li C. et al., 2016; Li et al., 2017f


*BDC, bladder cancer; BRC, breast cancer; CRC, colorectal cancer; ENDC, endometrial cancer; GC, gastric cancer; GMEC, glioma microvascular endothelial cancer; HCC, hepatocellular carcinoma; LC, lung cancer; MM, multiple myeloma; NSCLC, non-small cell lung cancer; OSTS, osteosarcoma; OVC, ovarian cancer; PNC, pancreatic cancer; PPTC, papillary thyroid cancer; PTC, prostate cancer; TNM, tumor node metastasis.*

## Autophagy-Inducing LncRNAs in Cancer

Majority of the autophagy-modulating lncRNAs have a positive relationship with the induction of autophagy. Hence, increase expression of these lncRNA in tumor induced autophagy and decrease expression of these lncRNA suppressed autophagy. Some examples are listed as follow.

### HOX Transcript Antisense RNA

HOX transcript antisense RNA contains 2158 nucleotides and was discovered by Rinn et al. (2007). It is located on intergenic space of HOXC11 and HOXC12 in chromosome 12q13.13 (Rinn et al., 2007). Abnormal expression of HOTAIR has been noticed for most of the cancers including, BT, BRC, CRC, GC, LVC, NSCLC, OVC, and PNC (Loewen et al., 2014; Zhou et al., 2014; Miao et al., 2016; Gerardo et al., 2017). Several research groups have reported HOTAIR association in different cancers evolutionary processes including, EMT, TNM, prognosis, drug resistance, metastasis, DFS, OS, and tumor development (Loewen et al., 2014; Zhou et al., 2014). Recently, a growing number of studies have revealed HOTAIR contexts in the regulation of autophagy, cancer progression, and drug resistance (Yang et al., 2016; Bao et al., 2017; Sun et al., 2017). Liu’s research group proved that, upregulated HOTAIR in HCC cells and tissues induce autophagy by promoting two major ATG3 and ATG7 (Yang et al., 2016). It is also proved that, HOTAIR expression increase along with STAT3 and ATG12 (key of autophagosome formation) through suppressing cancer suppressing micro RNA miR-454-3p in chondrosarcoma (Bao et al., 2017). Sun et al. (2017) revealed that HOTAIR abundancy in ENDC cells significantly induce autophagy which controls the development of chemo-resistance toward cisplatin through the expression of Beclin-1 and P-glycoprotein.

### Metastasis-Associated Lung Adenocarcinoma Transcript 1

Metastasis-associated lung adenocarcinoma transcript 1 is located on chromosome 11q13, containing over 8.7 kb nucleotides and was first identified in NSCLC since 2003 (Ji et al., 2003). It has been proven that MALAT1 plays significant roles in the development, proliferation, invasion, and metastasis of BDC, BRC, CRC, HCC, LC, NSCLC, and osteosarcoma (OSTS) (Ji et al., 2003; Gutschner et al., 2013; Hou et al., 2017; Li et al., 2017c; Zuo et al., 2017; Xiong et al., 2018). Up-regulated MALAT1 promote proliferation and metastasis of PTC cells and tissues (Li L. et al., 2016) via inducing autophagy. To facilitate the process, MALAT1 interacts with RNA binding protein HuR to activate autophagy via controlling post-transcriptional effects of cytotoxic granule-associated RNA binding protein TIA1 (Li L. et al., 2016). It has also been postulated that, aberrant expression of MALAT1 modulate autophagy in various cancers including glioma, GC, HCC, and RTB by controlling micro RNAs miR-216b, miR-101, miR-124, and miR-23b-3p (Yuan et al., 2016; Fu et al., 2017; Huang X. et al., 2017; YiRen et al., 2017). To maintain homeostasis of the cancer micro-environments, up-regulated MALAT1 induce conserved autophagy process directly or indirectly to take part in the progression of chemo-resistance and multi-drug resistance (Yuan et al., 2016; YiRen et al., 2017). More recently, YiRen et al. (2017) revealed that MALAT1 regulate GC progression and autophagy-mediated chemo-resistance via controlling micro RNA miR-23b-3p (YiRen et al., 2017). Gao D. et al. (2017) demonstrated that MALAT1 is highly expressed in MM along with HMGB1 to promote carcinogenesis by significantly expressing two key autophagy regulatory proteins LC3B and Beclin 1 (The mammalian ortholog of yeast ATG6). Their *in vivo* investigation suggests that knockdown of MALAT1 would be an effective target of MM growth inhibition by autophagy suppression (Gao D. et al., 2017).

### Plasmacytoma Variant Translocation 1

Plasmacytoma variant translocation 1 was first identified in murine leukemia virus-mediated T lymphomas. It contains 1716 nucleotides and is located on chromosome 8q24.21 (Zeidler et al., 1994). After its discovery, the roles of PVT1 have been identified in various cancers, including BDC, BRC, LC, MPM, and NSCLC (Cui et al., 2016; Li et al., 2017d; Lu et al., 2017; Zhang X.W. et al., 2017; Zhou et al., 2017; Guo et al., 2018; Li H. et al., 2018). Ma and coworkers proved that PVT1 is significantly up-regulate in GMEC and promote Atg7 and Beclin-1 expression. They reported that excessive endothelial cell proliferation and migration is mediated by PTV1/Atg7/Beclin-1 (Ma et al., 2017c). Huang and co-workers revealed that expression of PVT1 directly activates ULK1, an autophagy activating protein, in PDA) cells, patients sample, and *in vivo* xenograft model. PVT1 promotes pathogenesis by regulating miR-20a-5p (Huang et al., 2018). Thus, PVT1/ULK1/autophagy/miR-20a-5p may lead to being a novel therapeutic target of PDA.

### H19

Maternally expressed non-protein coding transcript H19 is lying on the imprinted region of chromosome 11p15.5 and it is 2.3 kb in length (Cui et al., 2002). H19 is transcribed by RNA polymerase II and dysregulation of H19 is associated with BRC, CRC, GC, Glioblastoma, HCC, HNC, LC and NSCLC (Hu et al., 2016; Matouk et al., 2016; Chen et al., 2017b; Luo et al., 2017). Brannan et al. (1990) first discovered H19 as a riboregulator. To date, numerous evidence has been established for H19’s association in various human cancers through distinctive molecular pathways (Chen T. et al., 2016;Chen et al., 2017b). Expression of H19 was found to be increased in both PPTC cells and tissues along with estrogen receptor β which may trigger autophagy through regulating ROS and ERK1/2 pathways. Higher expression of H19 promotes PPTC pathogenesis where further investigation may lead to better understanding of PPTC carcinogenesis through H19/autophagy regulation (Li M. et al., 2018).

### Others

Increasing number of autophagy-modulating lncRNAs are being identified but some of them attracted less attention. Wang et al. (2014) described, BANCR activate autophagy and contribute to proliferation and apoptosis of both PPTC cells and tissues. The overexpression of BANCR promote conversion of LC3-II/LC3-I, activated autophagy promote cells growth and reduce apoptosis in G1 phase (Wang et al., 2014). LncRNA-p21 is a hypoxia-responsive intergenic non-coding RNA which is highly expressed in hepatoma and glioma (Işın et al., 2015; Shen et al., 2017). Overexpression of lncRNA-p21 is associated with autophagy induction in hepatoma and glioma cells through HIF-1/Akt/mTOR/P70S6K pathways, resulting in excessive proliferation, motility, reduced apoptosis, and reduced radiosensitivity. Therefore, knockdown of lncRNA-p21 is a new target of radiotherapy as its knockdown potentially alter the molecular events and increase radiosensitivity of hypoxic tumor cells (Shen et al., 2017). HNF 1A-AS1 is located on chromosome 12. It is associated with larger tumor size, advanced TNM stage, excessive growth and apoptosis process of HCC cells and tissues through sponging tumor suppressor miR-30b-5p and inducing autophagy (Liu et al., 2016). Yu et al. (2018), recently noticed LCPAT1 lncRNA in LC which is directly regulated with autophagic flux. The overexpression of LCPAT1 and LC3 was found to be in both LC cells and tumor samples which accelerate the autophagic flux formation to promote carcinogenesis. Whereas, knockdown of LCPAT1 can significantly reduce *in vivo* tumor size by reducing LC3, ATG3, ATG5, ATG7, ATG12, ATG14, and Beclin1expression (Yu et al., 2018). Chen and co-workers shown that PTENP1 is a lncRNA which is downregulated in HCC and SB based hybrid baculovirus vectors mediated insertion of PTENP1 potentially work as targeted anti-tumor agent in HCC cells by reducing proliferation and migration by activating autophagy in PI3K/AKT pathways (Chen et al., 2015).

## Autophagy-Inhibiting LncRNAs in Cancer

Some lncRNA has an inverse relationship with autophagy and one example that has been studied extensively is MEG3. MEG3 is an imprinted gene which was first identified in 2000 (Miyoshi et al., 2000). It contains ∼1600 nucleotides and is found in 14q32.3 chromosomal position (Ma et al., 2018). Extensive research demonstrated that MEG3 expression is significantly reduced in cancer and it affects the proliferation, migration, and metastasis of most cancers including BRC, CRC, GC, glioma, HCC, LC, NSCLC, and PNC (Gong and Huang, 2017; He et al., 2017; Wang P. et al., 2017; Wei and Wang, 2017; Zhang C.Y. et al., 2017; Ma et al., 2018). Ying et al. (2013) speculated that MEG3 inversely regulate cellular autophagy process via the p53 pathways, and reduced MEG3 induce autophagy to promote BDC proliferation and progression. Down-regulation of MEG3 promote tumorigenesis and progression of epithelial OVC cells proliferation and colony formation through inhibiting autophagy process (Xiu et al., 2017). On the other hand, up-regulation of MEG3 inhibits the expression of autophagy-related proteins LC3, ATG3, and LAMP1 (Xiu et al., 2017). These findings led to the development of MEG3 as a potential biomarker of early diagnosis and treatment of OVC. More recently, Ma et al. (2017a) also proved that MEG3 is association with cisplatin-induced glioma cells death by regulating autophagy.

### Others

Jiang et al. (2018) explored a novel relationship of CASC2 in temozolomide (chemotherapy drug) resistance of glioma. CASC2 is negatively downregulated with miR-193a-5p in temozolomide resistant glioma tissues and induce autophagy by controlling mTOR expression to promote drug resistance (Jiang et al., 2018). Ma et al. (2017b) have described lncRNA AC023115.3 up-regulated in glioma cells after cisplatin treatment and induce cisplatin-mediated apoptosis by inhibiting autophagy process via miR-26a/GSK3β axis. Wang and colleagues showed that lncRNA CTA (CTA) is significantly downregulated in OSTS cells and cancer tissues in contrast with the adjacent normal tissues. CTA downregulated expression is also associated with the advanced TNM stage, larger tumor size and reduced chemosensitivity of doxorubicin through autophagy process (Wang Z. et al., 2017). Micro-RNA miR-210 is negatively regulated with CTA in OSTS and promote apoptosis of OSTS cells, whilst overexpression of CTA inhibit autophagy and sensitize to doxorubicin subsequently in both *in vitro* and *in vivo* (Wang Z. et al., 2017). PCA3 is a newly identified lncRNA, located on chromosome 9q21-22 and highly specific for PTC (Popa et al., 2007). He et al. (2016) reported that PCA3 is overexpressed in PTC to promote proliferation, migration, and invasion by sponging miR-1261 through inhibiting PRKD3 and blocking protective autophagy. On the other hand, silencing of PCA3 is able to induce protective autophagy and lessen the PTC progression which could be a novel target of personalized treatment (He et al., 2016). Shan’s team established that, silencing of lncRNA POU3F3 significantly reduce CRC cells proliferation, migration, and activate autophagy process by enhancing the expression of autophagy-related genes and proteins Beclin-1, ATG5, ATG7, and LC3 II which is novel therapeutic target of CRC (Shan et al., 2016).

## Autophagy-Modulating LncRNAs Either Induce or Inhibit Autophagy in Cancer

In the literature, we found some reports describing the same lncRNA but with opposite relationship with autophagy. Here, we include three examples which have been studied extensively.

### Highly Up-Regulated in Liver Cancer

Highly up-regulated in liver cancer was an lncRNA originally characterized in HCC as a significantly overexpressed lncRNA (Panzitt et al., 2007). HULC contains two exons, 1.6k nucleotides in length, located on chromosome 6p24.3. It is significantly dysregulated in most of the cancers including CRC, GC, HCC, OSTS and PNC (Panzitt et al., 2007; Chen et al., 2017c; Li et al., 2017g; Ma et al., 2017d; Shaker et al., 2017; Yu et al., 2017). A number of groups addressed HULC dysregulation and its molecular mechanisms in various cancer cells proliferation, migration, apoptosis, and metastasis but limited reports have focused on autophagy. Zhao et al. (2014) established that overexpression of HULC is clinically correlated with the developmental process of GC by promoted proliferation, migration, invasion, and reduced cellular apoptosis by inducing autophagy. Xiong et al. (2017) found that HULC overexpression induces autophagy and resulting in reduced chemosensitivity of potent chemo drugs 5-fluorouracil, oxaliplatin, and pirarubicin in HCC cells. Moreover, inhibition of protective autophagy by silencing of HULC sensitize these three drugs activity through controlling sirt1 protein in HCC (Xiong et al., 2017). In the contrary, Chen et al. (2017a) reported that HULC suppresses *in vitro* apoptosis and *in vivo* tumor development through autophagy blockage in epithelial ovarian carcinoma. Up-regulated HULC inhibits expression of ATG7, LC3-II, and LAMP1 while activates SQSTM1/p62 to promote carcinogenic events (Chen et al., 2017a).

### Growth Arrest-Specific 5

LncRNA GAS5 was first identified in 1988. It is a tumor suppressor lncRNA which contains 630 nucleotides and encoded at chromosome 1q25 (Schneider et al., 1988). So far, it is well-established that GAS5 plays key roles in diverse molecular functions in cancers (Pickard and Williams, 2015; Ma et al., 2016; Gao Q. et al., 2017). Meta-analysis of GAS5 shown that, it is associated with DFS, OS, LNM and tumor stages (I, II, III, IV) (Gao Q. et al., 2017). GAS5 is well-known for the negative regulation of most cancer cells survival (Song et al., 2014). Zhang and co-workers have demonstrated that down-regulating GAS5 would inhibit autophagy in NSCLC and facilitate drug resistance. Overexpression of GAS5 through vector mediated transfection induced autophagy and promote chemotherapy (cisplatin) response in NSCLC cells (Zhang et al., 2016). Gu et al. (2018) reported that GAS5 expression and autophagy were both downregulated in BRC cells and patients sample GAS5 expression is negatively correlated with depth tumor size, advanced TNM and poor prognosis of diseases. Interestingly, vector-mediated overexpression of GAS5 triggers autophagy and increases LC3, ATG3, and p62 expressions through sponging miR-23a. These findings may be developed into a targeted therapy for BRC through GAS5/miR-23a/ATG3 axis (Gu et al., 2018). However, another experiment conducted by Huo’s group also demonstrated reduced GAS5 expression in cisplatin-resistant glioma cell lines. Further investigations on the mechanisms have shown that GAS5 down-regulated glioma cells become resistant to cisplatin by increasing autophagosomes formation (Huo and Chen, 2018).

### Regulator of Reprogramming

The lncRNA ROR was first identified in pluripotent stem cells. It contains four exons, totally 2.6 kb in length and is located on chromosome 18q21.31 (Loewer et al., 2010; Zhan et al., 2016). ROR has been reported to be involved in isolated cellular processes, including growth, proliferation, migration, apoptosis, autophagy and metastasis of BRC, CRC, GC, HCC, NPC, and PNC cancers (Takahashi et al., 2014; Pan et al., 2016; Wang S. et al., 2016; Zhan et al., 2016; Li et al., 2017a; Peng et al., 2017; Wang Y. et al., 2017). Chen Y.M. et al. (2016) proved that ROR suppress autophagy and gemcitabine-induced cell death (apoptosis) in BRC cells by regulating miR-34a. In another study, however, Li’s team showed that ROR is up-regulated in PNC to promote basal autophagy which suppresses PKM2 and reduce chemo-sensitivity (gemcitabine) of cells (Li C. et al., 2016). Li et al. (2017f) also identified ROR overexpression reduce autophagy to increase proliferation, invasion, migration, and tamoxifen resistance in BRC cells and tissues. On the other hand, silencing of ROR effectively increases the sensitivity of tamoxifen, proliferation, and migration by inducing autophagy (Li et al., 2017f).

## Discussion

Advanced technologies have improved our understanding of the role of lncRNAs in cancer. At this stage, it is hard to consider lncRNAs as cancer biomarker because the sensitivity and specificity are still not at the desired level. However, researchers may consider using the autophagy-modulating lncRNAs for the development of biomarkers and targeted cancer therapy. From the growing research based knowledge on lncRNAs, autophagy and cancers, we may declare that most lncRNAs are involved in tumorigenesis through inducing or inhibiting the autophagy pathway.

Autophagy is a conserved regulatory process which is essential for both normal and cancer microenvironments under stress and hypoxic conditions (Xu et al., 2017). Advances in NGS technologies identified more than 3500 putative lncRNAs and the epigenetic roles of lncRNAs have been identified in many diseases including cancers. The discovery of lncRNAs and their molecular signals in cancers through autophagy process has drawn keen attention by scientists as biomarkers and targets for cancer therapy of lncRNAs/autophagy modulated diseases (Xu et al., 2017). However, autophagy and lncRNAs research is still in its fancy compared to its quantity, limited research methods of relationship establishment studies and inadequate therapeutic and clinical trials. Therefore, a major part is still under investigations. The effects of autophagy-modulating lncRNAs are very controversial from its discovery. Both autophagy and lncRNAs may accelerate the carcinogenesis or suppress the cancers (Wang et al., 2015; Liu et al., 2016; Lu et al., 2017; Ma et al., 2017b; Xu et al., 2017). In addition, the dual roles playing autophagy and lncRNAs made of difficult to comprehend the regulatory mechanisms in cancers (Zhang J. et al., 2017).

The regulatory process of autophagy composed of initiation, phagophore nucleation, autophagosome structure formation, and autolysosomal fusion to degrade the unwanted proteins or cytoplasmic components from the body (Mizushima, 2007). It is now well-established that lncRNAs participate in cellular regulatory autophagy pathways (Xu et al., 2017; Yang et al., 2017; Sun, 2018), for example, H19, BRCA1, MEG3, PTENP1, and MALAT1 are involved in initiation; ROR, loc146880, and AC023115.3 are involved in nucleation; TGFB2-OT1, GAS5, HNF1A-AS1, PCGEM1, and HULC are involved in elongation; Chast and MALAT1 are involved in lysosomal fusion process (Yang et al., 2017). With the perspective of ATGs genes, lncRNAs provided a new paradigm of gene expression in the autophagy pathway. An increasing number of lncRNAs have been identified in cancer which triggers autophagy to either promotion or suppression of carcinogenesis (Xu et al., 2017; Sun, 2018). It is now a precious research question that, does autophagy impact on the expression of lncRNAs to promote or suppress cancer? So far, PVT1 is the only reported lncRNA which is regulated by autophagy in diabetes (Li Z. et al., 2016). To address this question in cancer we are currently working on autophagy regulated lncRNAs and their impact on CRC pathogenesis.

## Conclusion and Insights

Cancer is the second leading cause of death worldwide. Incidences and deaths are increasing because of poor understanding of the disease, diagnostic techniques, and proper treatments. Progression of treatments remains dissatisfactory because most of the cancer patients were diagnosed after development of metastasis. Recently circulating lncRNAs has been considered for diagnostic and therapeutic purpose even though their mechanisms remain unclear (Qi et al., 2016; Sun, 2017). In this review, we have described the roles autophagy-regulated lncRNAs on cancer (Table 1), and their impact on autophagy regulation through the distinct network (Figure 3). At present, the research on the lncRNAs involvement on autophagy pathways is still in its primary stage. Although every day new lncRNAs are reporting on various cancer type, most of the researchs mainly focused on their impact on cancer pathogenesis. By considering the lncRNAs impact on autophagy mechanisms (Yang et al., 2017), more critical functions underlying autophagy are waiting to be demonstrated. Some autophagy-modulated lncRNAs described in this review are very specific with identical tissue types, for example HULC, PCA3, PVT1 may serve as a potential biomarkers and target therapy in identical cancer types (He et al., 2016; Ma et al., 2017c; Xiong et al., 2017). Therefore, extensive studies are needed to address the interaction between lncRNAs and the complex regulatory autophagy process with selective target genes and signaling transductions for the discovery of new targets, prognostic and diagnostic biomarkers and target therapy.

## Author Contributions

All authors have participated sufficiently in the work to take public responsibility for the content. We have worked together in the conception, drafting and revising the articles to provide an intellectual content of critical importance to the work described and final approval of the version to be published.

## Conflict of Interest Statement

The authors declare that the research was conducted in the absence of any commercial or financial relationships that could be construed as a potential conflict of interest.

## Abbreviations

ATGs: autophagy-related genes
BANCR: BRAF-activated long non-coding RNA
BDC: bladder cancer
BRC: breast cancer
BT: brain tumor
CASC2: cancer susceptibility candidate gene 2
CRC: colorectal cancer
CTA: LncRNA CTA
DFS: disease-free survival
EMT: epithelial–mesenchymal transition
ENDC: endometrial cancer
GAS5: growth arrest-specific 5
GC: gastric cancer
GMEC: glioma microvascular endothelial cancer
HCC: hepatocellular carcinoma
HMGB1: high-mobility group box 1 protein
HNC: head and neck cancer
HNF 1A-AS1: a natural antisense transcript of HNF 1A
HOTAIR: Hox transcript antisense RNA
HULC: highly up-regulated in liver cancer
LC: lung cancer
LCPAT1: lung cancer progression associated transcript 1
LEUK: leukemia
lncRNAs: long non-coding RNAs
LNM: lymphatic node metastasis
LVC: liver cancer
MALAT1: metastasis-associated lung adenocarcinoma transcript 1
MEG3: maternally expressed gene 3
MM: multiple myeloma
MPM: malignant pleural mesothelioma
ncRNAs: non-coding RNAs
NGS: next-generation sequencing
NSCLC: non-small-cell lung cancer
OS: overall survival
OSTS: osteosarcoma
OVC: ovarian cancer
PCA3: prostate cancer antigen 3
PDA: pancreatic ductal adenocarcinoma
PKM2: pyruvate kinase isozymes 2
PNC: pancreatic cancer
PPTC: papillary thyroid carcinoma
PRKD3: protein kinase D3
PTC: prostate cancer
PTENP1: pseudogene of tumor suppressor gene PTEN
PVT1: plasmacytoma variant translocation 1
ROR: regulator of reprogramming
RTB: retinoblastoma
SB: sleeping beauty
sirt1: silent information regulator 1
STAT3: signal transducer and activator of transcription 3
TNM: tumor node metastasis
